# Biosafety evaluation of BaSi_2_O_2_N_2_:Eu^2+^/PDMS composite elastomers

**DOI:** 10.3389/fbioe.2023.1226065

**Published:** 2023-07-07

**Authors:** Zheyuan Zhang, Mingrui Zong, Jinrong Liu, Jianing Ren, Xiaoming Liu, Ran Zhang, Jiayu Cui, Lingxiang Sun, Hao Song, Yanjie Zhang, Bing Li, Xiuping Wu

**Affiliations:** ^1^ School and Hospital of Stomatology, Shanxi Medical University, Taiyuan, Shanxi, China; ^2^ Shanxi Province Key Laboratory of Oral Diseases Prevention and New Materials, Taiyuan, Shanxi, China; ^3^ Research Institute of Photonics, Dalian Polytechnic University, Dalian, Liaoning, China

**Keywords:** mechanoluminescent, BaSi_2_O_2_N_2_:Eu^2+^, polydimethylsiloxane, composite, biosafety

## Abstract

In recent years, mechanoluminescent (ML) materials have shown great potential in stress sensing, mechanical energy collection and conversion, so they have attracted wide attention in the field of stomatology. In the early stage of this study, BaSi_2_O_2_N_2_:Eu^2+^ ML phosphors were synthesized by two-step high temperature solid state method, and then mixed with Polydimethylsiloxane (PDMS) in different proportions to obtain BaSi_2_O_2_N_2_:Eu^2+^/PDMS ML composites with different mass fractions (10%,20%,30%,40%,50%). Then its biosafety was evaluated by Cell Counting Kit-8 (CCK-8), Calcein-AM/PI fluorescence staining, hemolysis, oral mucosal irritation, acute and subacute systemic toxicity tests. The experimental results show that the biosafety of BaSi_2_O_2_N_2_:Eu^2+^/PDMS ML composite elastomers with different mass fraction is in line with the existing standards, and other related properties can be further studied.

## 1 Introduction

In recent years, a unique luminescence phenomenon—mechanoluminescent (ML) has attracted widespread attention (Wang et al., 2022), which first originated in 1605″Advancement of Learning” (Jha and Chandra, 2014). ML in a broad sense refers to all luminescence phenomena generated by various mechanical effects such as friction, extrusion, etc. In a narrow sense, it refers specifically to the luminescence caused by elastic deformation, plastic deformation or fracture deformation (Bai et al., 2021; Fujio et al., 2022). Since mechanical interactions permeate all aspects of life, ML could theoretically provide new solutions to challenging problems in biology, optoelectronics, and energy and environmental sciences (Xiong et al., 2021; Zhuang and Xie, 2021). In addition, it has received a lot of attention in dental materials science because of its demonstrated potential in stress sensing, mechanical energy harvesting and conversion (Shin et al., 2022).

Temporomandibular disorders (TMD) is one of the common diseases in oral clinic, and its pathogenic factors are various, including joint, masticatory muscle, occlusal and psychological disorders (Li and Leung, 2021), so it has always been an important and difficult point in the process of clinical diagnosis and treatment. Occlusal abnormalities are often considered by clinicians as a potential factor in TMD (Manfredini et al., 2017), and the relationship between them has always been concerned and controversial. Some scholars believe that the position of the condyle during apical malocclusion (intercuspalocclusion, ICO) determines the balance of the masticatory system, while the abnormal occlusal relationship may destroy this phenomenon, change the functional position of the mandible and the relationship between the condyle and the articular fossa, and then increase the risk of TMD (Ohrbach et al., 2020). Occlusal splint is a common and effective method for the treatment of abnormal occlusal function (Wieckiewicz et al., 2015). It is found that after stable occlusal splint treatment, abnormal muscle and joint activity in patients with TMD are significantly reduced, abnormal occlusal contact and mandibular movement are improved, and symptoms such as limited opening and joint pain are greatly solved (Al-Moraissi et al., 2020). However, some studies have shown that the existing occlusal materials have some shortcomings, such as high water absorption and solubility, easy to be affected by saliva and can not accurately reflect the actual occlusal situation (Grymak et al., 2022). Therefore, it is necessary to look for an alternative material which can provide accurate occlusal detection function and better comprehensive performance.

The emergence of ML materials provides a new idea for optimizing occlusal detection and analysis in oral clinic and realizing occlusal visualization. ML materials have good physical and chemical stability, and the luminous intensity is proportional to the stress (Bai et al., 2022). Among them, the nitrogen oxide fluorescent material BaSi_2_O_2_N_2_:Eu^2+^ (BSON:Eu^2+^) shows super ML (Seibald et al., 2012). After mechanical stimulation such as pressing and stretching, the green ML can last for tens of seconds. Because the material type is nitrogen oxide and the preparation process has no pollution to the environment (Yun et al., 2016), it can be mixed with flexible matrix polydimethylsiloxane (PDMS), which is widely used in clinic, to prepare ML composite elastomer, so as to establish the relationship between mechanical force and optical signal.

In this study, BaSi_2_O_2_N_2_:Eu^2+^ phosphors were synthesized by two-step high temperature solid state method to further improve their luminous intensity, and then mixed with PDMS to prepare a series of BaSi_2_O_2_N_2_:Eu^2+^/PDMS composite elastomers with different mass fraction. Medical biomaterials related to oral cavity need to be strictly tested by relevant regulatory agencies before they are used in clinic, in order to minimize the adverse effects caused by direct contact with tissues, including *in vitro* cell tests, animal tests and so on (Xie et al., 2017). Therefore, according to the ISO7405/ISO10993 standard, the biological safety of BaSi_2_O_2_N_2_:Eu^2+^/PDMS composite elastomer was preliminarily evaluated by Cell Counting Kit-8 (CCK-8) cytotoxicity test, Calcein-AM/PI fluorescence staining, hemolysis test, oral mucosal irritation test, subacute and acute systemic toxicity test, in order to explore the possibility of its clinical application and provide experimental basis for future clinical application.

## 2 Experimental sections

### 2.1 Preparation of materials

#### 2.1.1 Preparation of BaSi_2_O_2_N_2_:Eu^2+^ phosphors

**FIGURE 1:**
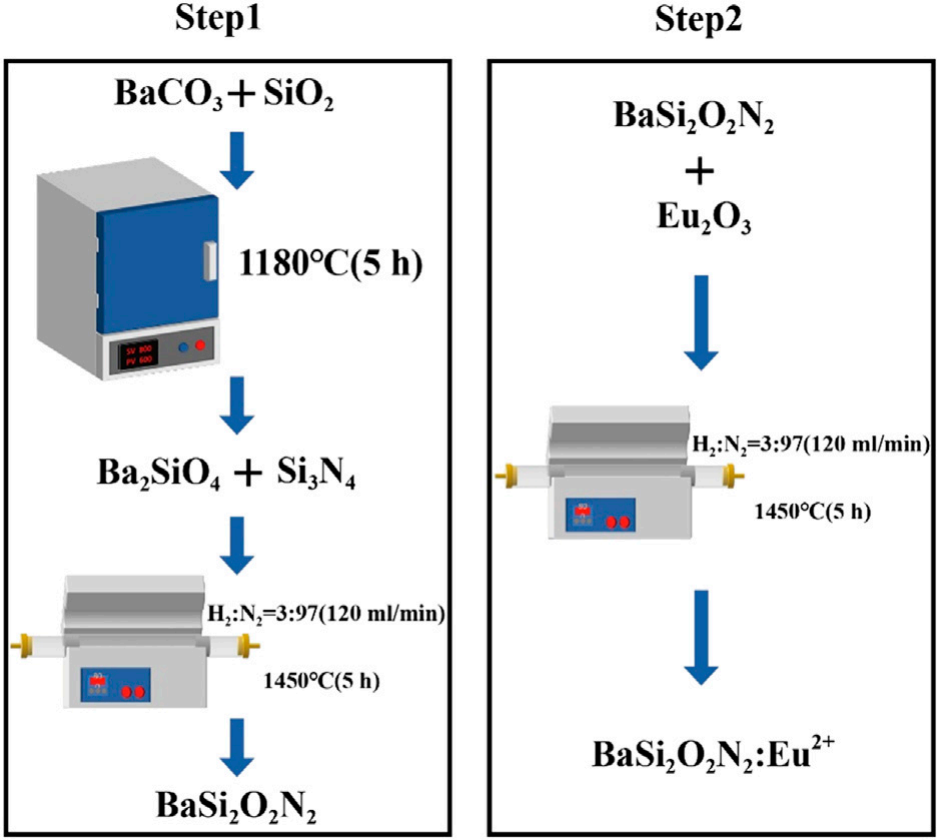
Synthesis of BaSi_2_O_2_N_2_:Eu^2+^ phosphors.

As shown in Figure 1, BaSi_2_O_2_N_2_:Eu^2+^ phosphors was prepared by two-step high temperature solid state method.

Step one: first, BaCO_3_ and SiO_2_ are ground and mixed and placed in a 1180°C box muffle furnace, sintered for 5 h and cooled, and Ba_2_SiO_4_ is obtained after re-grinding. Then add Si_3_N_4_, put it into a high temperature tube furnace after grinding and put into the reducing atmosphere with H_2_/N_2_ ratio of 3/97 (atmosphere flow rate is 120 mL/min). After sintering at 1450°C for 5 h, BaSi_2_O_2_N_2_ is obtained by cooling grinding.

Step two: add metal oxide Eu_2_O_3_ on the basis of the first step, grind it in a high temperature tube furnace and put it into a reducing atmosphere with a H_2_/N_2_ ratio of 3/97 (120 mL/min), sintering at 1450°C for 5 h, cooling and grinding to obtain BaSi_2_O_2_N_2_:Eu^2+^ phosphors, which is put into a sealed bag for subsequent performance characterization and testing.

#### 2.1.2 Preparation of BaSi_2_O_2_N_2_:Eu^2+^/PDMS composite elastomers

The core content of the process is to mix the luminescent material of the powder with the organic matrix. The organic matrix selected in this experiment is PDMS (composed of the main body and curing agent, the mixing ratio is 10:1). BaSi_2_O_2_N_2_:Eu^2+^ phosphors and PDMS were weighed at the mass ratio of 10%、20%、30%、40%、50%, mixed and stirred to get the ML complex, which was left for a period of time to eliminate bubbles, and then cured at room temperature for 24 h to obtain the target BaSi_2_O_2_N_2_:Eu^2+^/PDMS composite elastomer.

#### 2.1.3 Preparation of ML occlusal splint

The initial model of the occlusal plate was coated on the single jaw plaster tooth model, then the silicone rubber was coated on the outer layer, and the single jaw plaster dental mold covered with the splint was stripped off after the silicone rubber was cured. BaSi_2_O_2_N_2_:Eu^2+^/PDMS was injected into the silicone rubber, then the silicone rubber was covered with a single jaw plaster dental mold, and then cured at 80°C for 1 h.

### 2.2 Characterization

X-ray diffractometer (XRD) is used to observe the phase structure and crystal structure of BaSi_2_O_2_N_2_:Eu^2+^, and then field-emission scanning electron microscopy (FESEM) is usually used to detect the micro-morphology and observe the distribution of particles. The prepared ML composite elastomer was irradiated under ultraviolet lamp for 3–5 min, then in a completely dark environment, simulated tooth bite force was applied to it after the afterglow disappeared completely, and its ML Phenomenon was observed.

### 2.3 Preparation of the specimens and extracts

The material samples were uniformly made into long 4 mm, wide 5 mm and 1 mm thick, and the BaSi_2_O_2_N_2_:Eu^2+^/PDMS ML composite elastomers were washed in anhydrous ethanol for 20 min, then washed with distilled water, routinely sterilized, dried and sterilized by high pressure steam. Put it in normal saline (the ratio of the surface area of the specimen to the extraction medium is 6 cm^2^/mL) and put it in a water bath at 37°C for 72 h.

### 2.4 Biosafety tests

#### 2.4.1 Cytotoxicity test

##### 2.4.1.1 CCK-8 assay

L929 fibroblasts were resuscitated and passaged for 2–3 times, then Dulbecco’s modified Eagle medium (DMEM) culture medium containing 10% embryonic bovine serum was used to make single cell suspension (2.5×10^4^ cells/mL) and inoculated on 24-well plate (400 μL/well). Then the cells were incubated in a cell incubator of 37°C and 5%CO_2_ for 24 h. After observing the adhesion of the cells, the original medium was discarded and a new medium was added. The BaSi_2_O_2_N_2_:Eu^2+^/PDMS composite elastomers with the same specifications and different mass fractions (the experimental group) were clamped into a 24-well plate with ophthalmic tweezers, while the control group only added DMEM medium. Three 24-well plates were inoculated under the same conditions and cultured in cell incubator for 1, 3 and 5 days.

On the first, third and fifth day, 24-well plates were taken out, and the morphology of cells was observed under inverted microscope. 40 μL WST-8 solution was added to each well, and then transferred to 96-well plate (100 μL/well) after 1.5 h of incubation. The oplical densiy (OD) of each well was measured at 450 nm wavelength by enzyme-linked immunosorbent assay (Elisa), and the cell survival rate was calculated.

$$Cell\;Viability\left(\%\right)=\left(material\;group–blank\;group\right)/\left(control\;group–blank\;group\right)$$

##### 2.4.1.2 Calcein-AM/PI fluorescence staining

Calcein-AM/PI double staining method was used to stain living cells and dead cells, in which Calcein-AM only stained living cells because it could easily penetrate the living cell membrane, hydrolyze in the cytoplasm and emit strong green fluorescence, while PI only stained dead cells because it could not pass through the living cell membrane and could pass through the disordered region of the dead cell membrane to reach the nucleus and embedded into the DNA double helix of the cell to produce red fluorescence. The method of culturing cells and adding materials was the same as 2.4.1.1.24-well plates were taken out on the first, third and fifth day, respectively, and the morphology of cells was observed under inverted microscope. 100 μL Calcein-AM was added to each 10 mL medium, and 20 min–25 min was incubated at 37°C. After that, 20–200 μL PI solution was added to each 10 mL medium, and 5 min was stained at room temperature. Finally, live cells (yellow-green fluorescence) and dead cells (red fluorescence) were detected by fluorescence microscope at 490 ± 10 nm; in addition, only dead cells could be observed in 545 nm.

#### 2.4.2 Oral mucosa irritation test

The subjects were 25 healthy golden gophers aged about 5 months, and every 5 gophers were set as an experimental group. There was no abnormality in bilateral buccal mucosa. The buccal mucosa of one side of each golden gopher was sutured with medical sutures to fix the BaSi_2_O_2_N_2_:Eu^2+^/PDMS composite elastomers of the same size, and the contralateral buccal mucosa was used as a blank control without any treatment. Every day after operation, whether the specimen fell off, whether the mucosa around the specimen was abnormal, such as hyperemia, swelling and erosion, and whether the animals had signs of poisoning and death were observed. Two weeks later, the golden gophers were killed under excessive anesthesia, and the specimens were removed. The mucosa and surrounding tissues of the contact and contralateral parts were embedded, fixed and sliced, and stained with hematoxylin-eosin (HE) for histopathological observation.

#### 2.4.3 Subacute systemic toxicity test

60 healthy SD rats weighing about 130 g were randomly divided into 6 groups (5 experimental groups, 1 control group, half male and half female). They adapted to the laboratory environment 7 days in advance and had to fast overnight without water before the experiment. The rats in the experimental group were perfused with BaSi_2_O_2_N_2_:Eu^2+^/PDMS composite elastomers extract (10%、20%、30%、40%、50%) with the dose of 50 mL/kg, while the rats in the control group were perfused with the same dose of normal saline. Once a day for 28 days, and then observed for 7 days. During this period, the signs of poisoning and death were observed, and the food utilization rate and relative growth rate of body weight were calculated by measuring weekly food consumption and weight gain of rats. At the end of the experiment, the animals were killed under excessive anesthesia, and the important tissues and organs were selected for dissection to observe the abnormal changes of each tissue and organ.

$$relative\;growth\;rate\;of\;body\;weight\;\left(\%\right)=weight\;gain\;\left(g\right)\;/\;initial\;weight\;\left(g\right)\times 100\%$$

$$food\;utilization\;rate\;\left(\%\right)=weight\;growth\;\left(g\right)\;/\;total\;food\;consumption\;\left(g\right)\times 100\%$$

#### 2.4.4 Acute systemic toxicity test

60 healthy C57 mice with about 17 g were randomly divided into 6 groups (5 experimental groups, 1 control group, half male and half female). The mice in the experimental group were intravenously injected with BaSi_2_O_2_N_2_:Eu^2+^/PDMS composite elastomers extract (10%, 20%, 30%, 40%, 50%) at the dose of 50 mL/kg every day, while the control group was injected with the same dose of normal saline. The symptoms of collapse, cyanosis, dyspnea, abdominal irritation, diarrhea and tremor were observed immediately after injection. The general behavior, clinical toxic symptoms and death were observed at 4, 24, 48 and 72 h after injection. at the same time, the experimental mice were weighed and recorded, and the body weight changes of mice were monitored. 72 h later, the animals were killed under excessive anesthesia, and the important tissues and organs were dissected to observe the abnormal changes of each tissue and organ.

#### 2.4.5 Hemolysis test

Heart puncture took 6-week-old New Zealand rabbit whole blood 10mL, add 2% potassium oxalate normal saline solution 0.5 mL anticoagulation, and then add normal saline diluted at 4:5. The experimental group was 5 g of different mass fraction of BaSi_2_O_2_N_2_:Eu^2+^/PDMS composite elastomers (10%, 20%, 30%, 40%, 50%), respectively, adding 10 mL 0.9% normal saline, the negative control group was 10 mL 0.9% normal saline, and the positive control group (complete hemolysis) was 10 mL distilled water. The test tube was placed in a 37°C water bath for 30 min (3 parallel samples in each group). Diluted fresh anticoagulant rabbit blood 0.2 mL was added and kept warm for 60 min. The supernatant OD value of the supernatant was measured by spectrophotometer 576 nm and the hemolysis rate was calculated after centrifugation at 758 *g* for 5 min.

$$Hemolysis\;rate\;\left(\%\right)=\left[\left({OD}_{X}-{OD}_{Z}\right)\;/\;\left({OD}_{Y}-{OD}_{Z}\right)\right]\times 100\%$$

in which OD_X_ is the experimental group (BaSi_2_O_2_N_2_:Eu^2+^/PDMS composite elastomers with different mass fractions), OD_Y_ is the positive control group (10 mL distilled water), and OD_Z_ is the negative control group (10 mL 0.9% saline).

## 3 Results and discussion

### 3.1 Characterization

**FIGURE 2:**
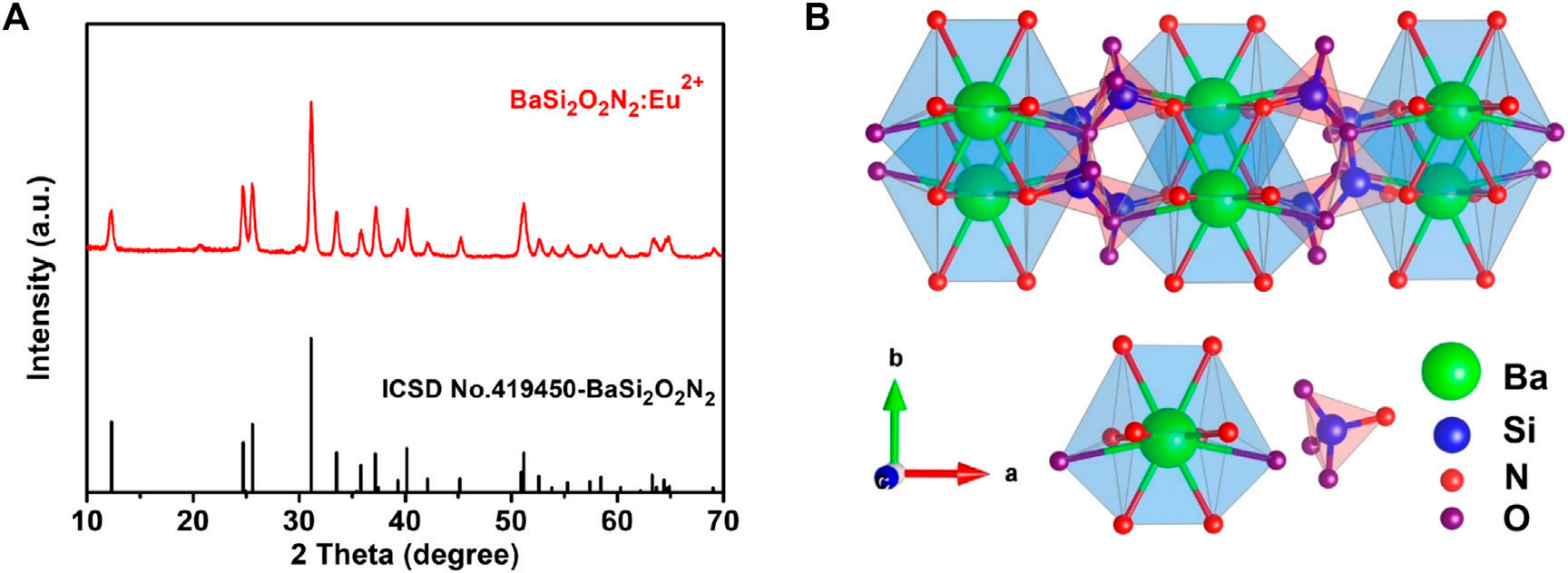
XRD patterns of BaSi_2_O_2_N_2_:Eu^2+^ **(A)** and Crystal structure of BaSi_2_O_2_N_2_ **(B)**.

Figure 2A is the X-ray diffraction pattern of BaSi_2_O_2_N_2_:Eu^2+^ phosphor and BaSi_2_O_2_N_2_ matrix prepared by two-step high temperature solid state method. From the diagram, we can see that all the diffraction peaks of BaSi_2_O_2_N_2_:Eu^2+^ prepared by this method are consistent with the standard data of BaSi_2_O_2_N_2_ crystal (ICSD number 419450). No other impurity phases are found. Eu^2+^ ions are effectively doped into the BaSi_2_O_2_N_2_ matrix lattice to form BaSi_2_O_2_N_2_:Eu^2+^ phosphors. Figure 2B shows the crystal structure of BaSi_2_O_2_N_2_ with space group Pbcn. BaSi_2_O_2_N_2_ is formed by periodic stratification of (Si_2_O_2_N_2_)^2-^ anions and Ba^2+^ cations. Ba^2+^ cations exist at only one crystal site, which coordinates with eight O atoms and two long N atoms to form a cube structure; (Si_2_O_2_N_2_)^2-^ anions are composed of high-density SiON_3_ tetrahedrons with a common vertex.

**FIGURE 3:**
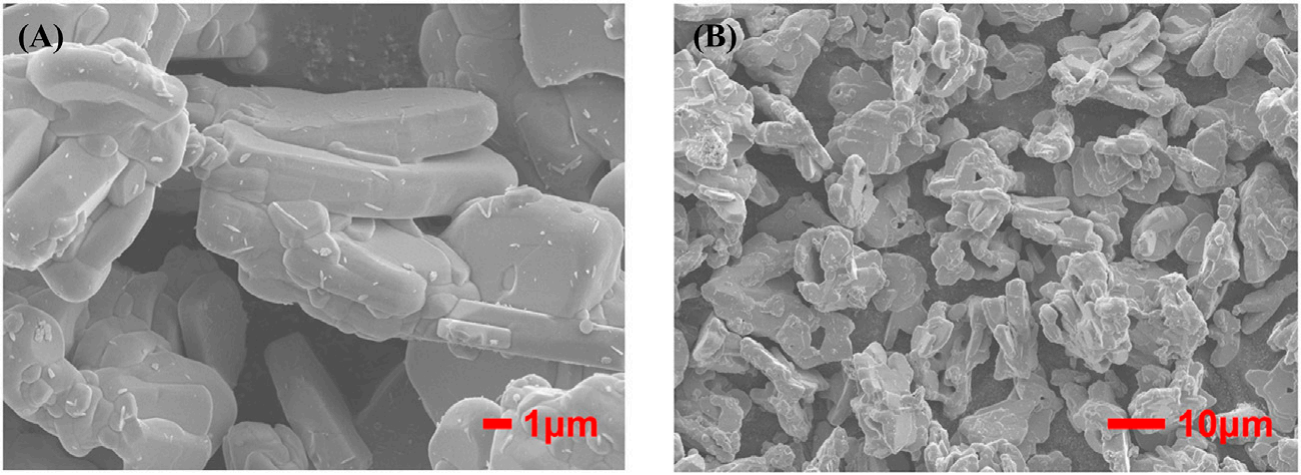
High **(A)** and low **(B)** magnification FESEM images of BaSi_2_O_2_N_2_:Eu^2+^.

Figures 3A, B are FESEM images of phosphors with different magnification respectively, showing clear layered structure of BaSi_2_O_2_N_2_, smooth surface, good particle dispersion and cubic phase consistent with crystal structure. Therefore, BaSi_2_O_2_N_2_:Eu^2+^ phosphors were synthesized by two-step high temperature solid state method, which laid a foundation for the preparation of ML composite elastomers.

**FIGURE 4:**
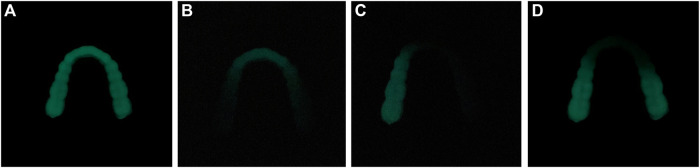
ML in different parts of dentition. **(A)** ML of the whole dentition **(B)** ML of anterior teeth **(C)** ML of left posterior teeth **(D)** ML of bilateral posterior teeth.

The ML occlusal splint emits cyan fluorescence after being stressed in the dark environment. It can be seen from Figures 4A–D that different parts of the dentition produce ML after stress. This phenomenon can guide us to find the site of abnormal occlusion as soon as possible and carry out the next step of targeted treatment.

### 3.2 Cytotoxicity test

#### 3.2.1 CCK-8 assay

**FIGURE 5:**
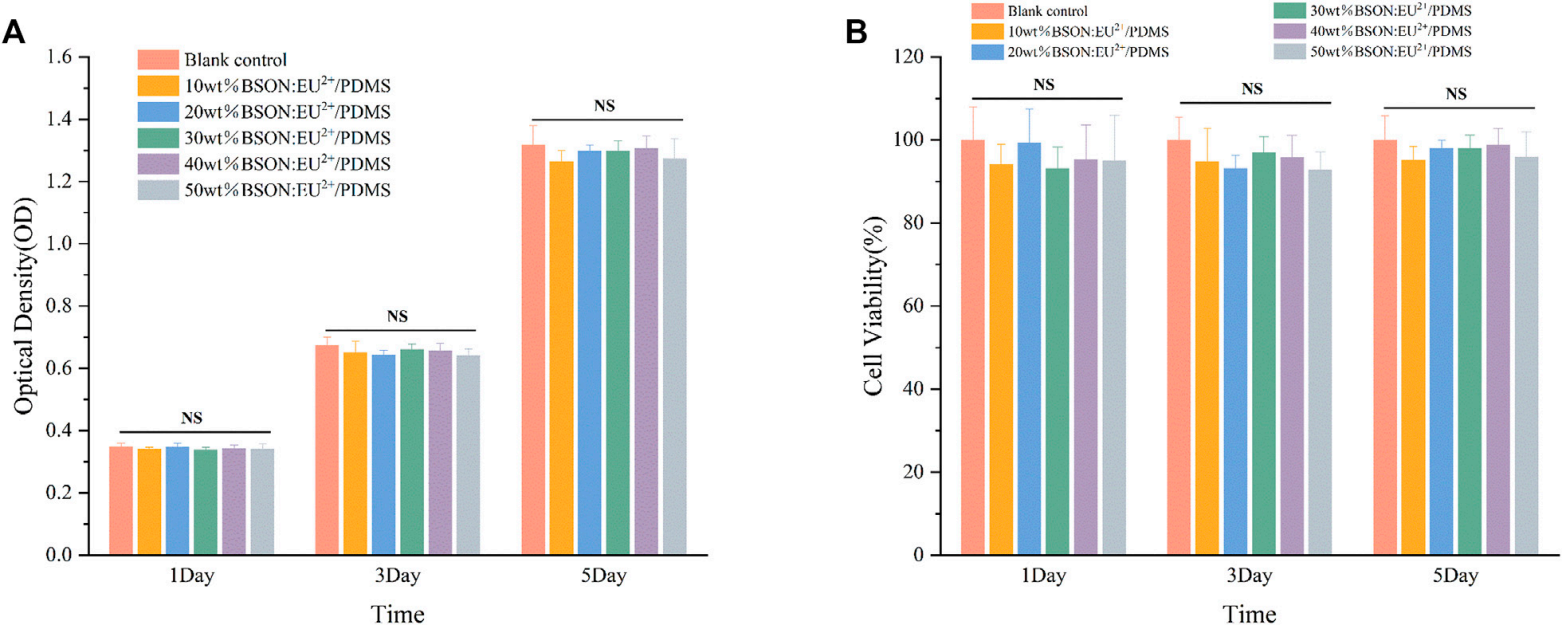
OD values **(A)** and cell viability **(B)** of each group.

After 1, 3 and 5 days of culture, the cells in each group were observed under inverted microscope, and the growth and morphology of cells in the blank control group and each experimental group were normal. The OD values and cell viability of each group were shown in Figure 5. The cell viability of each group was more than 90%, and the cytotoxicity grade (CTG) was 0. There was no significant difference between the experimental group and the blank control group and between different experimental groups (*p* > 0.05). The results showed that different mass fractions of BaSi_2_O_2_N_2_:Eu^2+^/PDMS composite elastomers had no cytotoxicity.

#### 3.2.2 Calcein-AM/PI fluorescence staining

**FIGURE 6:**
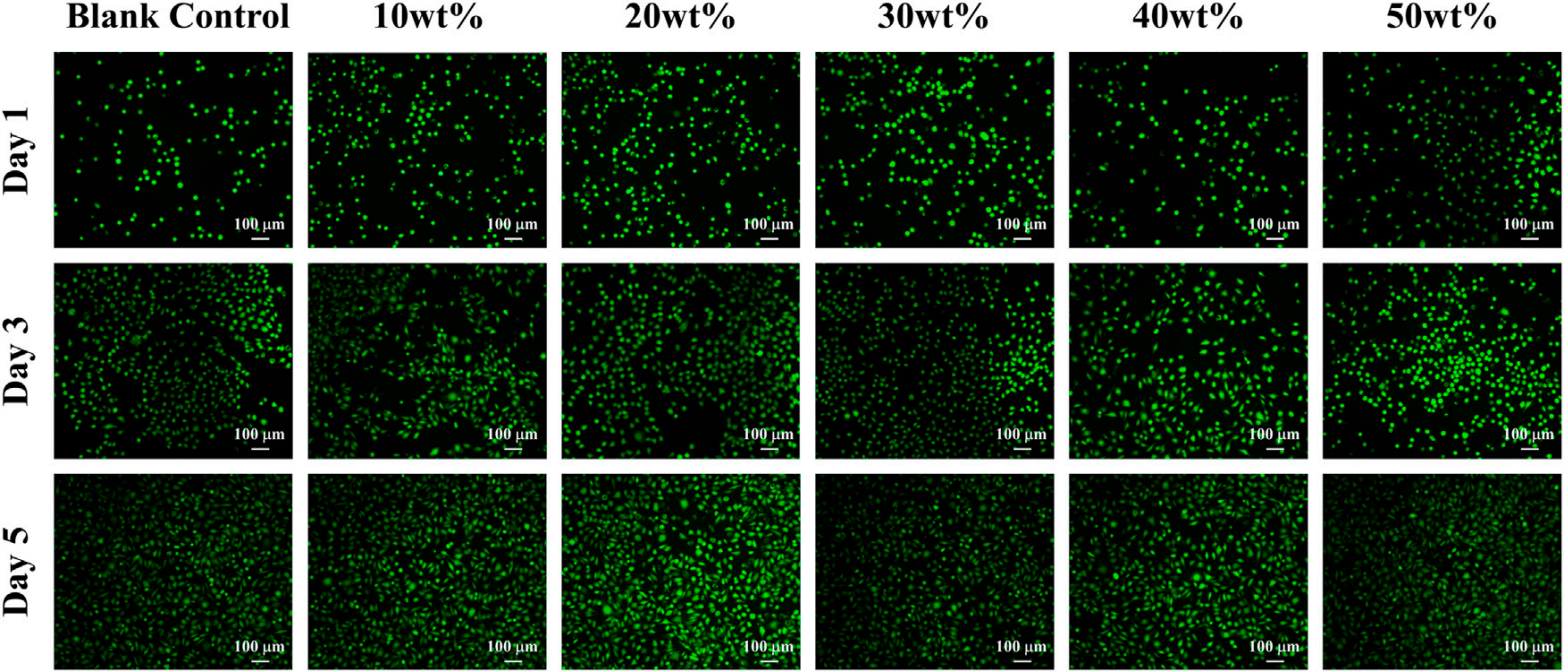
Calcein-AM/PI fluorescence staining of each group.

As shown in Figure 6, after Calcein-AM/PI fluorescence staining, most of the cells in the experimental group and control group showed green on the first, third and fifth day, and the cells survived well and the morphology was normal, which indicated that the mass ratio of 10%、20%、30%、40%、50% BaSi_2_O_2_N_2_:Eu^2+^/PDMS composite elastomers did not cause obvious harm to the cell activity.

The main methods of cytotoxicity assessment include CCK-8 assay and Calcein-AM/PI fluorescence staining to evaluate whether the cytocompatibility of materials meets the needs of biomaterials by measuring the effects of toxic substances on the morphological number, metabolic activity and proliferation of cultured cells (Cai et al., 2019; Sun et al., 2022; Dzedulionyte et al., 2023). Lmur929 fibroblasts were selected in the above two experiments, which have the advantages of strong reproductive ability, sensitivity to changes in environmental factors, and timely response to toxic substances in the materials (Lendahl et al., 2022).

The CCK-8 method is based on WST-8 (2-(2-methoxy-4-nitrophenyl)-3-(4-nitrophenyl)-5-(2-methoxy-4-disulfonated phenyl)-2H-tetrazolium monosodium salt) in the presence of electronic carrier 1-methoxy-5-methylphenazinonium sulfate dimethyl ester (1-MethoxyPMS), which is reduced by dehydrogenase in mitochondria to orange-yellow methyl Zan products with high water solubility. The more cells proliferate, the faster the color, the darker the color; the greater the cytotoxicity, the lighter the color (Kawano et al., 2019). For the same cells, the color is directly proportional to the number of living cells, and the absorbance at 450 nm wavelength can be measured by enzyme-linked immunosorbent assay (Elisa), which can indirectly reflect the activity of cells. In addition, according to the 10993–5 standard of the International Organization for Standardization, the cell survival rate is more than 90%, indicating that the material has no cytotoxicity.

The principle of Calcein-AM/PI fluorescence staining experiment is that Calcein-AM can only stain living cells, because it can easily penetrate the living cell membrane, hydrolyze in the cytoplasm and emit strong green fluorescence; PI only staining dead cells, because it can not pass through the living cell membrane, can pass through the disordered region of the dead cell membrane to reach the nucleus, and embedded into the DNA double helix of the cell to produce red fluorescence (Xue et al., 2019; Ke et al., 2020). It is worth mentioning that the premise of effective staining is that the changes in the activity of the corresponding cell model are physical and biochemical characteristics such as changes in esterase activity and plasma membrane integrity, and cytotoxic events that do not affect these cell characteristics may not be accurately evaluated by this method.

### 3.3 Oral mucosa irritation test

During the experiment, it was observed that the specimens of all groups of golden gophers were in good retention, normal behavior and mental state, without any abnormal conditions or adverse reactions. In addition, no adverse reactions such as redness, swelling, erosion and ulcer were found at the contact site of the specimen and its surrounding tissue.

**FIGURE 7:**
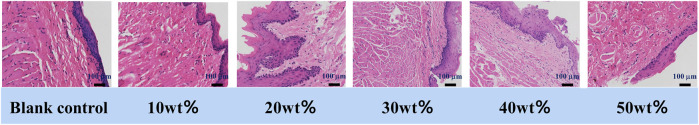
Histological observation of the oral mucosa.

Figure 7 shows the histological observation of oral mucosa in the experimental groups and the control group. The microscopic observation shows that compared with the blank control group, the cell morphology and structure of buccal mucosa and its surrounding tissue in each experimental group are normal, there is no epithelial hyperplasia, and the cells are well-delaminated and uniformly arranged. There is no inflammatory.

Cell infiltration in connective tissue, no hyperkeratosis, granular layer changes or other adverse changes. As the occlusal splint has been in direct contact with the oral mucosa for a long time, there is a certain degree of dissolution in the process (Reyes-Sevilla et al., 2018), so it is required that the material itself and tissue contact will not produce toxic substances. Oral mucosal irritation test is used to evaluate the irritation of materials to oral mucosa by suturing and fixing biomaterials in animals. In this experiment, after the buccal mucosa of golden gophers were exposed to BaSi_2_O_2_N_2_:Eu^2+^/PDMS composite elastomers with different mass fraction, no abnormal reaction of color, morphology and texture was observed in the buccal mucosa and its surrounding tissue, indicating that all of them had good biosafety.

### 3.4 Subacute systemic toxicity test: oral route

**FIGURE 8:**
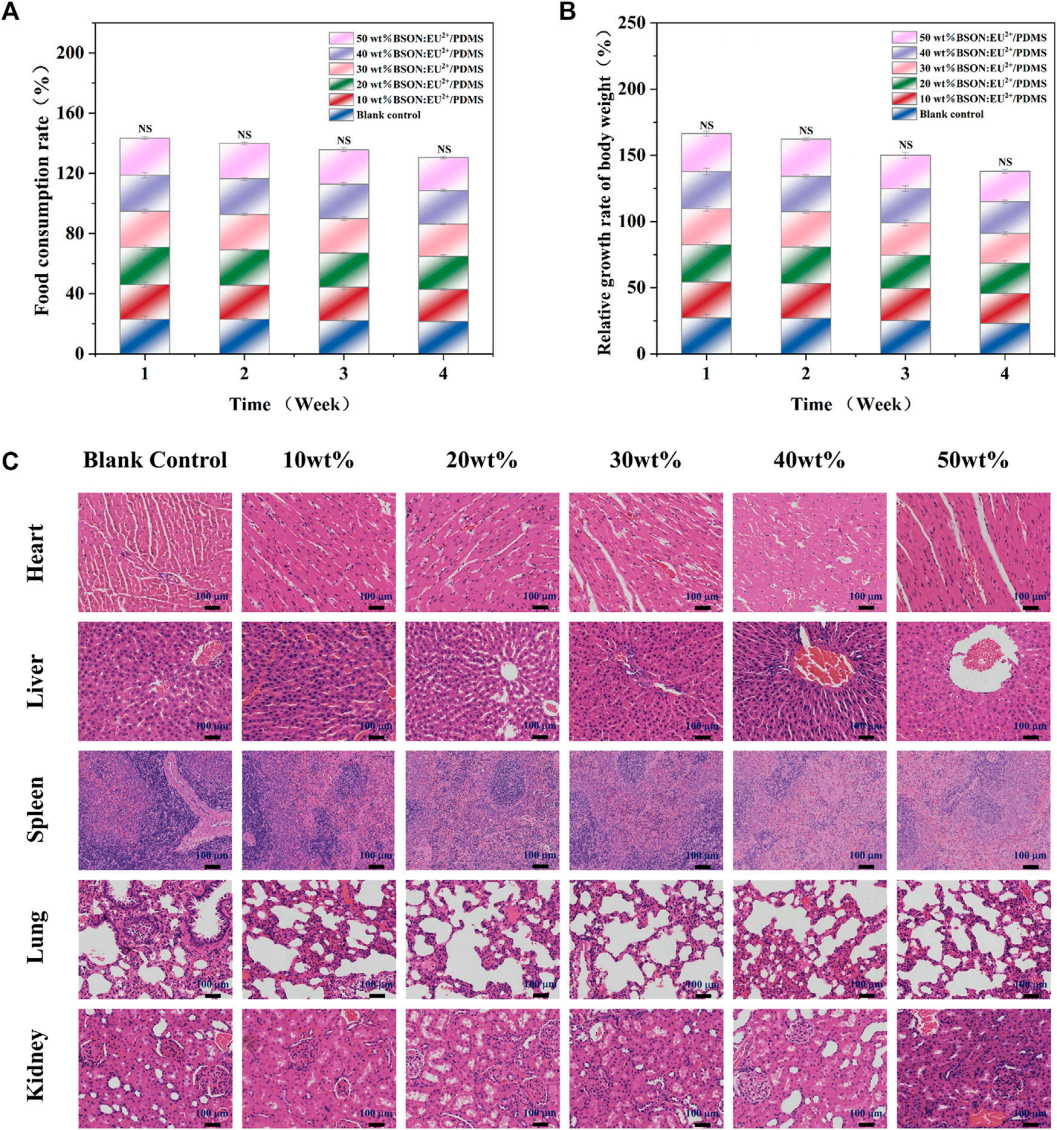
The relative growth rate of body weight **(A)** and food utilization rate **(B)** of SD rats and HE staining for the subacute systemic toxicity test of each group **(C)**.

During the experiment, it was observed that the behavior and mental state of all SD rats were normal, and there were no abnormal conditions or adverse reactions. According to the daily body weight changes and food consumption, the relative growth rate of body weight and food utilization rate could be calculated (Figures 8A, B). There was no significant difference between the experimental group and the control group and between different experimental groups by test of variance. In addition, compared with the control group, the important organs of the experimental group were normal in shape and size, and there were no abnormal changes; histopathological examination (Figure 8C) showed no pathological changes such as atrophy, degeneration or pigmentation, and no inflammatory cell infiltration.

Occlusal splint is in contact with human teeth and periodontal tissue for a long time and bears a variety of long-term stress. Its chemical composition and small substances may enter the human body along with the digestive tract, resulting in toxic effects and harm to tissues and organs of the whole body. Therefore, through the method of intragastric administration, we can ensure that enough drugs enter the digestive tract, simulate the way of toxic substances into the human body to a large extent, and evaluate the biosafety of the materials by observing the clinical symptoms and histological manifestations of animals (Okado et al., 2015).

### 3.5 Acute systemic toxicity test

**FIGURE 9:**
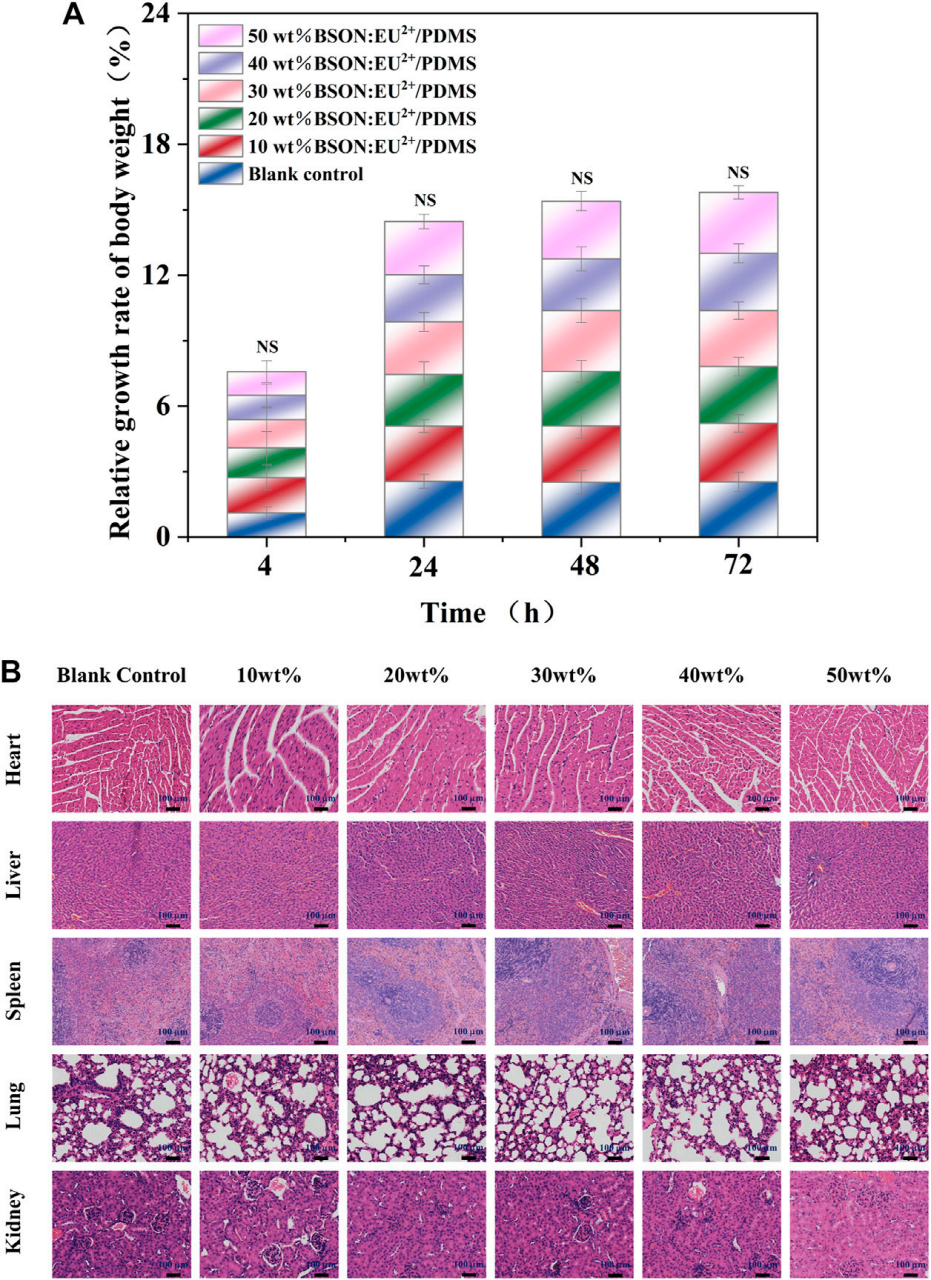
The relative growth rate of body weight of C57 mice **(A)** and HE staining for the acute systemic toxicity test of each group **(B)**.

After injection of material extract, there were no symptoms such as collapse, dyspnea, cyanosis, diarrhea and tremor. Within 72 h, the mice in the experimental group and control group moved freely, had a normal appetite, and had no clinical toxic symptoms or death. There was no significant difference in the relative growth rate of body weight of C57 mice between the experimental group and the control group and between different experimental groups (Figure 9A). There were no abnormal pathological changes such as inflammatory cell infiltration in the tissues and organs of mice in the experimental group (Figure 9B). The acute systemic toxicity test is different from the mucosal irritation test, which is not to detect the effect on the contact site, but to evaluate whether the material is potentially toxic to the whole body tissues and organs. If more than one mouse is abnormal during the experiment, such as dyspnea or even death or significant weight loss (more than 2 g), the material is proved to be toxic (Pillai et al., 2017). In this experiment, no abnormality was found in mice, and there was no death or weight loss in mice, indicating that the material has good biological safety.

### 3.6 Hemolysis test

**FIGURE 10:**
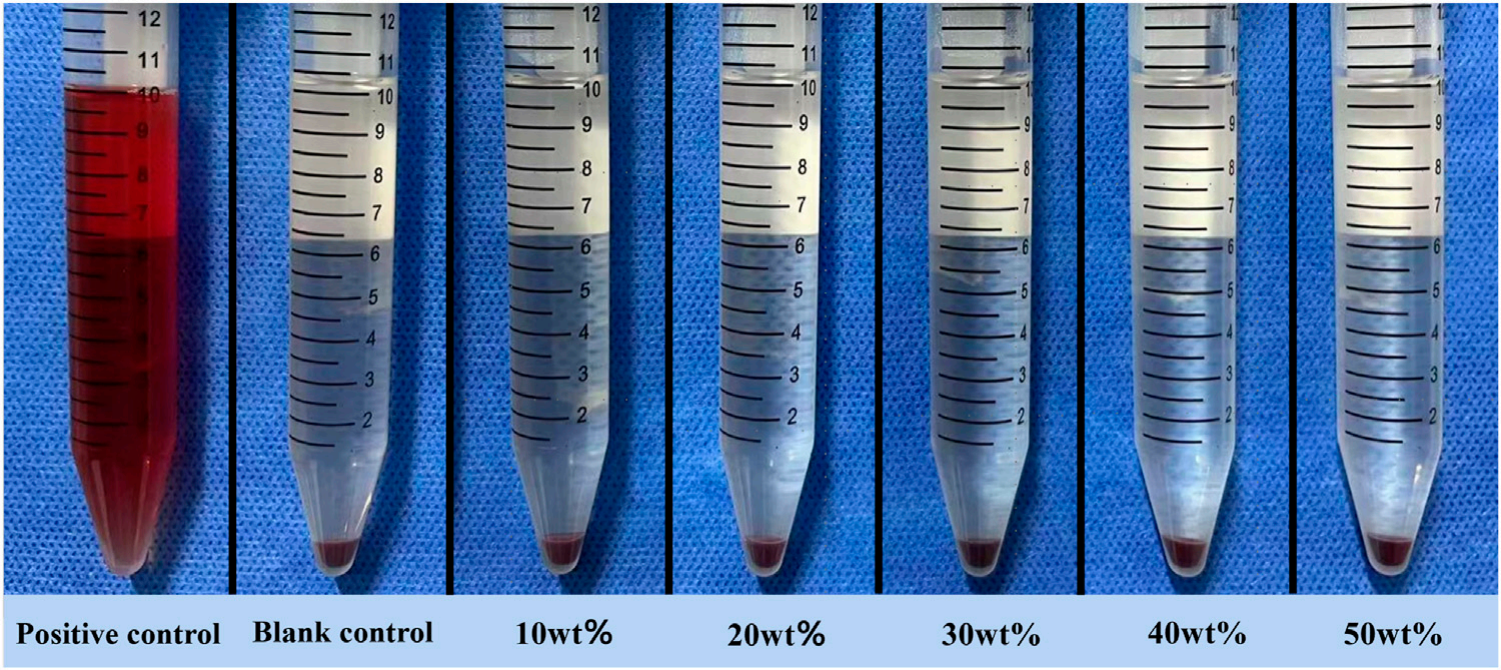
Hemolysis test supernatant.

The experimental results are obvious (Figure 10). A large number of red blood cells in the positive control group were destroyed until the supernatant was red, while the supernatant in the negative control group and each experimental group was clear and transparent, and almost no red blood cells were destroyed. The hemolysis rate of each experimental group was less than 5% (Table 1). There was no significant difference between the experimental group and the control group and each experimental group by variance test.

**TABLE 1:** OD values of the hemolysis test.

Groups	OD value			Average OD value	Hemolysis ratio (%)
	1	2	3		
**Positive control**	1.3425	1.3902	1.2843	1.3390	-
**Blank control**	0.0196	0.0212	0.0187	0.0198	-
**10 wt%BSON:EU** ^**2+**^ **/PDMS**	0.0314	0.0303	0.0313	0.0310	0.8465
**20 wt%BSON:EU** ^**2+**^ **/PDMS**	0.0295	0.0329	0.0314	0.0313	0.8667
**30 wt%BSON:EU** ^**2+**^ **/PDMS**	0.0311	0.0318	0.0291	0.0307	0.8213
**40 wt%BSON:EU** ^**2+**^ **/PDMS**	0.0325	0.0316	0.0293	0.0311	0.8566
**50 wt%BSON:EU** ^**2+**^ **/PDMS**	0.0301	0.0316	0.0295	0.0304	0.8010

The principle of hemolysis test is that if the test material is toxic, it will destroy the erythrocyte membrane and release hemoglobin in red blood cells. According to ISO10993-4, if the hemolysis rate of the material is less than 5%, it is proved to be non-toxic and meets the standard of biomedical application (Liu et al., 2022). In this experiment, the hemolysis rate of each experimental group is far less than the standard value, indicating that the material has good blood compatibility.

## 4 Conclusion

The ML phosphor BaSi_2_O_2_N_2_:Eu^2+^, was prepared by two-step high temperature solid state method, and then mixed with matrix PDMS to obtain ML composite elastomer—BaSi_2_O_2_N_2_:Eu^2+^/PDMS. The biocompatibility of BaSi_2_O_2_N_2_:Eu^2+^/PDMS composite elastomers with different mass fractions (10%, 20%, 30%, 40%, 50%) was evaluated by CCK-8 assay and Calcein-AM/PI fluorescence staining at the cellular level, acute and subacute systemic toxicity test, oral mucosal irritation test and hemolysis test. The results showed that the biosafety of different mass fractions of BaSi_2_O_2_N_2_:Eu^2+^/PDMS composite elastomers met the current standards, and other properties could be tested in the next step. For example, elasticity, wear resistance, solubility, stability and so on, select the best quality ratio, with a view to clinical application, to provide a more efficient method for occlusal visualization.

## Data Availability

The original contributions presented in the study are included in the article/supplementary material, further inquiries can be directed to the corresponding authors.
